# Effect of Butyrate on Collagen Expression, Cell Viability, Cell Cycle Progression and Related Proteins Expression of MG-63 Osteoblastic Cells

**DOI:** 10.1371/journal.pone.0165438

**Published:** 2016-11-28

**Authors:** Mei-Chi Chang, Yi-Ling Tsai, Eric Jein-Wein Liou, Chia-Mei Tang, Tong-Mei Wang, Hsin-Cheng Liu, Ming-Wei Liao, Sin-Yuet Yeung, Chiu-Po Chan, Jiiang-Huei Jeng

**Affiliations:** 1 Biomedical Science Team, Chang Gung University of Science and Technology, Kwei-Shan, Taoyuan City, Taiwan; 2 Department of Dentistry, Chang Gung Memorial Hospital, Taipei, Taiwan; 3 Graduate Institute of Clinical Dentistry and Department of Dentistry, National Taiwan University Hospital and National Taiwan University Medical College, Taipei, Taiwan; 4 Wei-Cheng International Dental Group, Taipei, Taiwan; Taipei Medical University, TAIWAN

## Abstract

**Aims:**

Butyric acid is one major metabolic product generated by anaerobic Gram-negative bacteria of periodontal and root canal infection. Butyric acid affects the activity of periodontal cells such as osteoblasts. The purposes of this study were to investigate the effects of butyrate on MG-63 osteoblasts.

**Methods:**

MG-63 cells were exposed to butyrate and cell viability was estimated by 3-(4,5-dimethylthiazol-2-yl)-2,5-diphenyltetrazolium bromide (MTT) assay. The mRNA and protein expression of type I collagen and cell cycle-related proteins were measured by reverse-transcriptase polymerase chain reaction (RT-PCR), western blotting or immunofluorescent staining. Cellular production of reactive oxygen species (ROS) was analyzed by 2',7'-dichlorofluorescein (DCF) fluorescence flow cytometry.

**Results:**

Exposure to butyrate suppressed cell proliferation, and induced G2/M (8 and 16 mM) cell cycle arrest of MG-63 cells. Some cell apoptosis was noted. The mRNA expression of cdc2 and cyclin-B1 decreased after exposure to butyrate. The protein expression of type I collagen, cdc2 and cyclin B1 were decreased, whereas the expression of p21, p27 and p57 was stimulated. Under the treatment of butyrate, ROS production in MG-63 cells markedly increased.

**Conclusions:**

The secretion of butyric acid by periodontal and root canal microorganisms may inhibit bone cell growth and matrix turnover. This is possibly due to induction of cell cycle arrest and ROS generation and inhibition of collagen expression. These results suggest the involvement of butyric acid in the pathogenesis of periodontal and periapical tissue destruction by impairing bone healing responses.

## Introduction

Microorganisms are shown to play important roles in the diseased processes of periodontal and pulpal/periapical lesions. Various periodontal and root canal pathogens such as *Porphyromonas*, *Eubacterium*, *Fusobacteria* and *Peptococci* etc. may be involved in the initiation and propagation of these diseased processes by generation a number of toxic products such as lipopolysaccharide, short chain fatty acids (SCFA), proteases etc. [1–5]. During the metabolism of amino acids, hexose or pentose by microorganisms, significant amounts of butyric acid are produced in the periodontal pockets and root canals [2–5], and affect the biological activities of adjacent periodontal cells (e.g., gingival fibroblasts, bone cells, periodontal ligament cells). The concentration of SCFAs (e.g., acetic acid, propionic acid and butyric acid) in gingival crevicular fluid (GCF) from diseased periodontal pocket is generally at mM concentration, and associated with the severity of periodontal diseases. SCFA levels of GCF declined after non-surgical periodontal treatment [4,6]. The mean concentrations of butyric acid in GCF collected from sites of severe periodontitis, mild periodontitis and healthy teeth are about 2.6 mM, 0.2 mM and undetectable, respectively [4]. The other paper also shows the level of butyric acid to be 0.5–16 mM in GCF from sites with different diseased status [7].

Butyrate at higher concentrations may inhibit leukocyte apoptosis and function, but stimulates leukocyte cytokine production. It also impedes the growth of vascular endothelial cells, gingival epithelial cells and fibroblasts [3,8,9]. Higher concentration of butyrate (1 mM) suppresses the Runt-related transcription factor 2 (Runx2), osterix, distal-less homeobox 5 (Dlx5), Msh homeobox 2 (Msx2), alkaline phosphatase (ALP), osteocalcin, and bone sialoprotein expression, but stimulates AJ18 expression of ROS17/2.8 osteoblasts [10], suggesting inhibition of differentiation. Butyric acid further suppressed the proliferation and Con A-stimulated interleukin 2 (IL-2), IL-4, IL-5, IL-6, and IL-10 production in splenic-T cells [11]. All these effects are involved in the diseased processes of periodontal and periapical tissue injuries.

ROS are critical molecules for induction of signal transduction and toxic events by chemicals and carcinogenic agents [12,13]. Recent study suggests that increased ROS levels are associated with bony destruction in periodontitis [14]. Butyrate has been shown to suppress the proliferation of periodontal tissue cells and thus contribute to the periodontal tissue inflammation and breakdown. The cell growth is tightly controlled by cell cycle and cell cycle-related genes such as cdc2, p21 and cyclins [15,16]. We hypothesized that butyrate may impair bone tissue healing via inhibition of collagen formation, cell growth and cell cycle progression of osteoblasts, inducing ROS production and involved in the pathogenesis of periodontal and periapical diseases. We therefore investigated the effect of butyrate on the growth, cell cycle progression, collagen expression and ROS production of MG-63 osteoblastic cells.

## Materials and Methods

### Materials

MG-63 osteoblastic cells were from American Type Culture Collection (ATCC, USA). All cell culture biologicals were obtained from Gibco (Life technologies, Grand Island, NY, USA). Propidium iodide (PI), sodium butyrate, 3-(4,5-dimethylthiazol-2-yl)-2,5-diphenyltetrazolium bromide (MTT) and 2’,7’- Dichlorodihydrofluorescein diacetate (DCFH-DA) were bought from Sigma (Sigma Chemical Company, St. Louis, MO, USA). The SuperScript TM III First-Strand DNA synthesis system for reverse transcriptase polymerase chain reaction (RT-PCR) was from Invitrogen (Invitrogen Corporation, Carlsbad, CA, USA). RNase A for flow cytometric analysis was from Becton-Dickinson (San Jose, CA, USA). Antibodies against Glyceraldehyde 3-phosphate dehydrogenase (GAPDH), cdc2, cyclin B1, p21, and type I collagen were obtained from Santa Cruz (Santa Cruz, USA). Antibodies for p27 and p57 were from GeneTex (GeneTex International Corporation, Hsin-Chu City, Taiwan).

### Culture of MG-63 cells

MG-63 cells were cultured in Dulbecco’s modified Eagle’s medium (DMEM) containing 10% fetal bovine serum (FBS), 1x penicillin and 100 μg/ml of streptomycin.

### Cell Viability Assay

Viability of cells was estimated by the MTT colorimetric assay. MG-63 cells (5 x 10^4^ cells/well) were cultured in 6-well culture plates for 24 h at 37°C. Cells were subsequently cultured in fresh medium containing different concentrations of butyrate (1–16 mM) for 5 days. Medium was aspirated and the insoluble formazan generated by viable cells were dissolved in dimethylsulfoxide (DMSO) and read against solvent blank (DMSO) at a wavelength of 540 nm (OD540) by an enzyme-linked immunosorbant assay (ELISA) reader (Multiskan Spectrum, USA) [17].

### Effect of butyrate on the cell cycle progression of MG-63 cells

In brief, 5 x 10^5^ of MG-63 cells were inoculated onto 6-well culture plates. After 24 h of cell adhesion, they were incubated to various concentrations of butyrate (1–16 mM) for 24 h. After incubation for 24 h, floating cells in the culture medium were collected. The attached MG-63 cells were detached from the culture wells by treatment with trypsin/ethylenediamine tetraacetic acid (EDTA). Thereafter we collected both the floating and attached cells. Cell cycle analysis was done as described previously [18,19]. Briefly, we washed the collected cells with phosphate buffered saline (PBS) and then fixed the cells in 70% ice-cold ethanol. After 24 h, the cells were washed with PBS, and then incubated in PBS containing RNase. Finally, the cells were stained for 15 min with propidium iodide (PI, 40 μg/ml). After washing and centrifugation, the fluorescence of PI in MG-63 cells was counted by flow cytometric analysis (FACSCalibur, Becton Dickinson, Worldwide Inc., San-Jose, California). The wavelength of laser excitation was set at 488 nm with an emission wavelength collected at higher than 590 nm. The PI fluorescence of 20000 cells was analyzed for both control and experimental samples. We determined the percentage of cells residing in sub-G0/G1, G0/G1-, S- and G2/M phases by using ModiFit software and CellQuest programs.

### Effects of butyrate on cell cycle-related genes expression

#### RNA isolation

In brief, 1–1.5 x 10^6^ of MG-63 cells were seeded into 10-cm culture dishes. Cells were allowed for attachment for 24 hours and then exposed to various concentrations of butyrate (0–16 mM) for 24 h. Total RNA was isolated with RNA isolation kit [20].

Reverse transcriptase–polymerase chain reaction (RT-PCR) was performed using specific primers for beta-actin (BAC), cdc2, cyclinB1, and p21 as described before [9,21]. Briefly, 3 μg of denatured total RNA was reverse transcribed in a total volume of 10 μl reaction mixture comprising 4 μl of random primer (500 μg/ml), 1 μl of dNTP (2.5 mM), 2 μl of 10x RT buffer, 1 μl of RNase inhibitor (40 U/μl) and 1 μl of RT (21 U/μl) at 42°C for 50 min. Then, we used 2 μl of cDNA for PCR amplification in a reaction volume of 50 μl comprising 5 μl of 10x Super TAQ buffer, 200 μmol of dNTP (2.5 mM), 1 μl of each specific primer, and 0.2 μl of Super TAQ enzyme (2 U/μl). The PCR reaction was performed at 94°C for 5 min for the first cycle, and then further amplified for 20–35 cycles at 94°C for 30 s, 55°C for 1 min and then 72°C for 30 s with a thermal cycler (Perkin Elmer 4800, PE Applied Biosystems, Foster city, CA, USA). Lastly, the reaction was completed at 72°C for additional 10 min. The specific primer pairs of this study were: cyclin B1, cdc2 and p21 as described before [9,21]. The PCR-amplified products were subjected to 1.8% agarose gel electrophoresis and then the gels were stained with ethidium bromide and pictures were taken. The PCR-amplified DNA product that presented linear in relation to the input RNA was used for picture and data presentation. The amplification of the β–actin (BAC) gene was used as control.

### Effects of Butyrate on Cell cycle-related, and Type I Collagen Protein Expression

Briefly, 1–1.5 x 10^6^ of MG-63 cells were inoculated onto 10-cm culture dishes. After 24 h of cell attachment, they were exposed to different concentrations of butyrate (0–16 mM) for 24 h. After washing with PBS, cells were disrupted in lysis buffer (10 mM Tris-HCl, pH 7; 140 mM sodium chloride; 3 mM magnesium chloride; 0.5% NP-40; 2 mM phenylmethylsulfonyl fluoride; 1% aprotinin; and 5 mM dithiothreitol) [17,18,20]. Then aliquots (20–50 μg protein) of cell lysates were subjected to 12.5% sodium dodecyl sulfate-polyacrylamide gel electrophoresis (SDS-PAGE) and conveyed to a polyvinylidene fluoride (PVDF) membrane. The membrane was then blotted with anti-human cdc2, cyclin B1, p21, p27, p57, type I collagen and GAPDH antibodies for 2 h. The membranes were then incubated with anti-goat, anti-mouse, or anti-rabbit horseradish peroxidase-linked secondary antibodies, respectively (Jackson ImmunoResearch Laboratories, West Grove, PA, USA) for 1 h. After washing of the membrane by buffer, ECL reagents (Amersham) were added and the chemiluminescence was identified by exposure of the membranes to Fuji films for 30 s to 10 min. The strength of immunoreactive band of GAPDH was utilized as control.

### Immunofluorescent Microscope Observation of Cellular Type I Collagen Expression

Briefly, 1 x 10^5^ of MG-63 cells were seeded on the sterile coverslips in a 24-well plate in DMEM and 10% FBS. After 24 h, they were subjected to different concentrations of butyrate (0–16 mM) for additional 24 h. Medium was decanted, and cells were rinsed with PBS and fixed for 20 min in 4% paraformaldehyde. Cells were washed by PBS, membrane penetration with 2% Triton X-100, incubated for 20 min in 0.3% v/v H_2_O_2_. After washed with PBS, cells were blocked in 5% bovine serum albumin (BSA) for 1 h and then incubated in primary antibodies (against type I collagen) at room temperature overnight. After washing by PBS, cells were incubated in corresponding secondary antibody in the dark for 1 h and counterstained for 30 min with 4',6-diamidino-2-phenylindole (DAPI, 1:1000) [17]. Finally the samples were mounted and observed/photographed by an Olympus IX71 inverted microscope and DP Controller/Manager software (Olympus Corporation).

### Effect of butyrate on cellular ROS levels

Briefly, 2.5 x 10^5^ of MG-63 cells were plated into 6-well culture wells. After 24 h of cell adhesion, cells were incubated in fresh medium containing different concentrations of butyrate (0–16 mM) for 24 h. ROS levels in MG-63 cell were measured by single cell DCF fluorescence flow cytometric analysis as described previously [19,22]. Briefly, MG-63 cells were treated with 10 μM DCFH-DA for the final 30 min. Cells were then washed with PBS, collected and soon subjected to flow cytometry analysis of cellular DCF fluorescence (Becton Dickinson, USA).

### Statistical Analysis

Four or more separate experiments were performed with similar results. Results were analyzed by one-way ANOVA and post-hoc Turky test. A p value < 0.05 was considered to have statistically significant difference between 2 groups. PCR and western blotting images were analyzed by Image J software for quantification and results were expressed as fold of control. In some experiments, the 50% inhibitory concentration of butyrate was calculated by regression analysis.

## Results

### Effect of Butyrate on the Proliferation of MG-63 Cells

**Fig 1:**
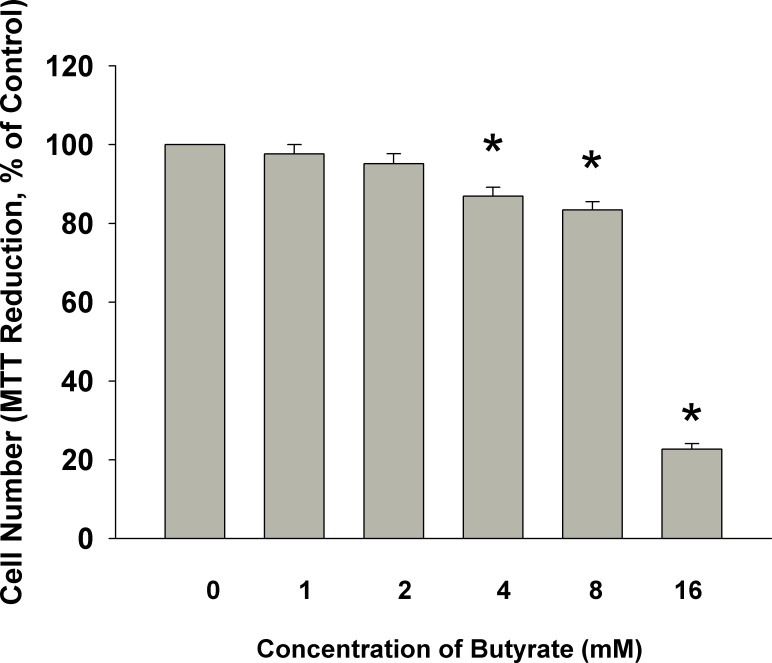
Effect of butyrate on the cell viability of MG-63 cells. MG63 cells were exposed to various concentrations of butyrate for 5 days. *denotes significant difference when compared with control (P < 0.05).

After exposure of MG-63 cells to different concentrations of butyrate (1–16 mM) for 5 days, evident growth inhibition of butyrate (4–16 mM) toward MG-63 cells was noted. Butyrate inhibited viable cells by 17% and 77%, respectively, at concentrations of 8 and 16 mM **(Fig 1)**.

### Effect of Butyrate on Cell Cycle Progression of MG-63 Cells

**Fig 2:**
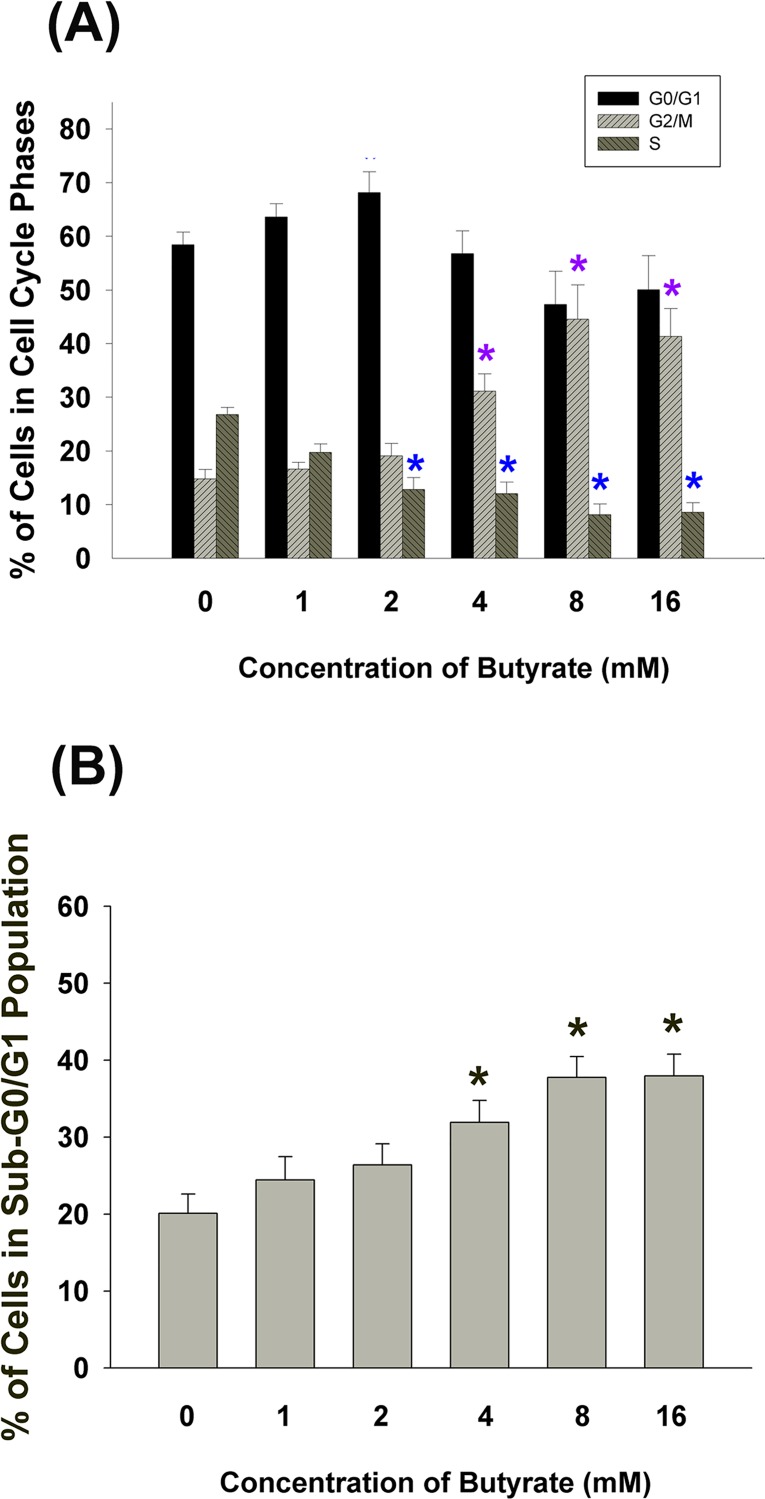
Effect of butyrate on cell cycle distribution of MG-63 cells. **(A)** The effect of different concentration of butyrate (0–16 mM) on G0/G1, G2/M, and S phase of MG-63 cells (5 x 10^5^ cells/well) after 24 hrs exposure time. *denotes significant difference when compared with control (P < 0.05). **(B)** The effect of different concentration of butyrate (0–16 mM) on sub G0/G1 population of MG-63 cells (5 x 10^5^ cells/well) after 24 hrs. *denotes significant difference when compared with control (P < 0.05).

Butyrate induced G2/M cell cycle arrest of MG-63 cells at concentrations of 4–16 mM **(Fig 2A)**. When MG-63 cells (5 x 10^5^ MG-63 cells/well) were exposed to butyrate (4–16 mM) for 24 h, a discernible increase in sub-G0/G1 population of MG-63 cells was observed **(Fig 2B)**.

### Effect of Butyrate on Cell cycle-related Gene Expression

**Fig 3:**
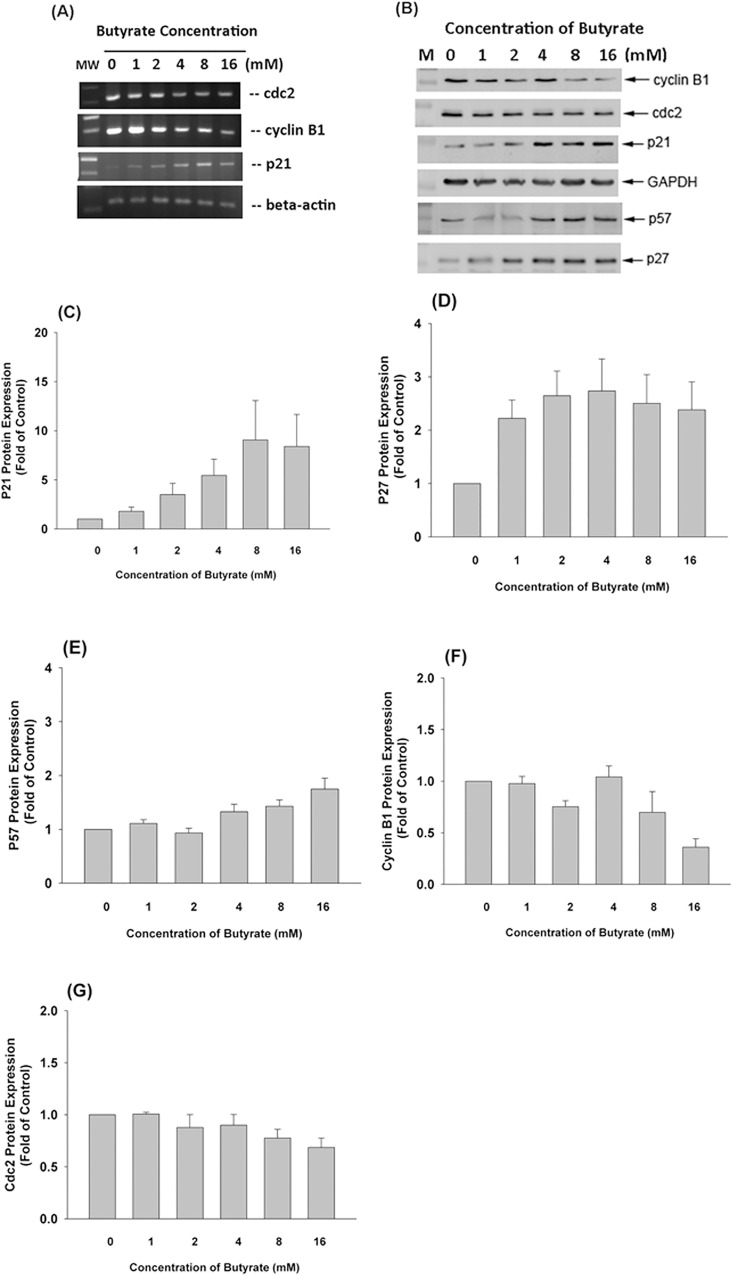
Effect of butyrate on cell cycle related genes and protein expression of MG-63 cells. **(A)** Effect of butyrate on cell cycle related genes (cdc2, cyclinB1, and p21) expression in MG-63 cells. MG-63 cells were exposed to different concentration of butyrate (0–16 mM) for 24 hours. Total RNA was isolated and used for RT-PCR analysis of cellular gene expression. Expression of β–actin was used as control, **(B)** Effect of butyrate on cell cycle related proteins (cdc2, cyclin B1, p21, p27 and p57) expression in MG-63 cells. MG-63 cells were exposed to butyrate (1–16 mM) for 24 hours. Equal amount of proteins from cell lysates were used for western blotting. One representative western blotting picture was shown.

RT-PCR analysis revealed a decline of cdc2, cyclinB1 mRNA expression and a rise in p21 mRNA expression was noted after exposure of MG-63 cells to butyrate (> 2 mM) **(Fig 3A)**.

### Effect of Butyrate on Cell Cycle- Related Proteins Expression

Similarly a decline in cdc2 and cyclin B1 protein expression of MG-63 cells was noted after exposure to butyrate (> 1 mM). An increase in p21, p27 and p57 protein expression was noted at butyrate concentrations higher than 2 mM **(Fig 3B)**. Quantitatively, butyrate stimulated the expression of p21 to 1.8–9.1-folds of control at concentrations ranging from 1–16 mM **(Fig 3C)**. At similar concentrations, butyrate also induced p27 protein expression to 2.22- to 2.73-fold of control **(Fig 3D)**. Accordingly, butyrate stimulated p57 proteins expression by 1.3 to 1.7-fold of control, at concentrations of 4–16 mM **(Fig 3E)**, Butyrate inhibited cyclin B1 protein expression with an IC50 of about 12.5 mM **(Fig 3F)**. Butyrate suppressed the cdc2 expression at concentrations of 8 and 16 mM, with 23% and 32% of inhibition **(Fig 3G)**.

### Effect of Butyrate on Type I Collagen Expression

**Fig 4:**
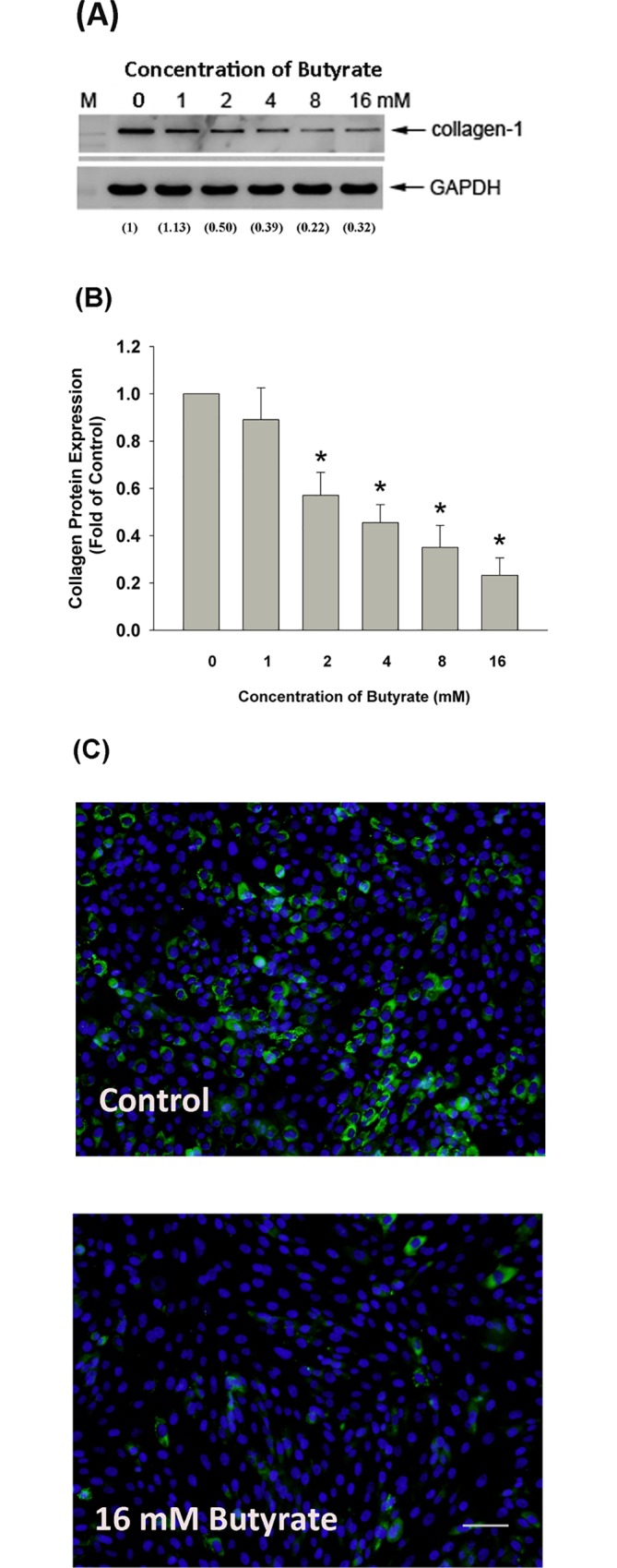
Effect of butyrate on type I collagen protein expression of MG-63 cells. **(A)** MG-63 cells were exposed to butyrate (1–16 mM) for 24 hours. Equal amount of proteins from cell lysates were used for western blotting. One representative western blotting picture was shown. Immunofluorescent analysis of type I collagen protein expression in MG-63 cells after treatment by 16 mM butyrate (control:0 mM), **(B)** Quantitative analysis for the effect of butyrate on collagen protein expression of MG-63 cells. Results were expressed as fold of control (as 1). *denotes statistically significant difference when compared with control. **(C)** Immunofluorescent analysis of type I collagen protein expression in MG-63 cells after treatment by 16 mM butyrate (control:0 mM), scale bar = 200 μm.

The protein level of type I collagen also decreased after exposure to butyrate (>1 mM) as analyzed by western blotting **(Fig 4A).** Quantitatively, butyrate inhibited the collagen protein expression of MG63 cells by 43% to 77% at concentrations ranging from 2 mM to 16 mM **(Fig 4B, S1 Table)**, with a 50% inhibitory concentration (IC50) about 3.2 mM. Accordingly, immunofluorescent analysis of type I collagen expression in MG-63 cells also revealed the decreased protein expression of type I collagen (green fluorescence) after exposure of MG-63 cells to butyrate **(Fig 4C).**

### Effect of Butyrate on ROS Production of MG-63 cells

**Fig 5:**
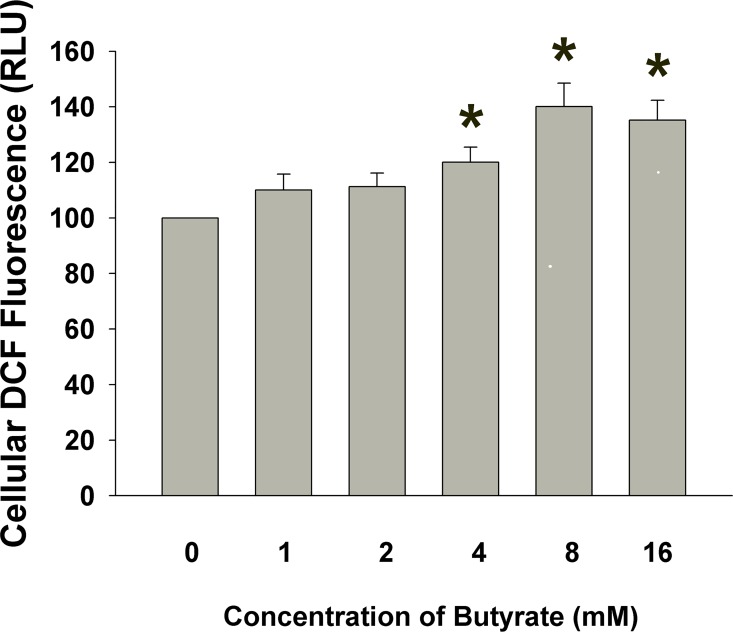
Effect of butyrate (1–16 mM) on cellular ROS level of MG-63 cells. One representative histogram of DCF fluorescence in control MG-63 cell and MG-63 cells exposed to 1–16 mM butyrate. An increase in DCF fluorescence was noted, indicating an increase of ROS production. *denotes statistically significant difference when compared with untreated control (as 100) (P < 0.05).

A 24-hr exposure of MG-63 cells to butyrate (4–16 mM) markedly elevated the cellular DCF fluorescence by 20–40%. As indicated in the representative histogram, 4–16 mM of butyrate stimulated the ROS levels of MG-63 cells **(Fig 5)**.

## Discussion

Microorganisms are the key etiologic factors of chronic periodontitis and pulpal/periapical diseases. Butyric acid and other SCFA are the metabolic/toxic products generated by numerous pathogenic microorganisms. Their levels in GCF may reach mM concentrations and pose toxic effect on periodontal and periapical tissue cells [3,4,7]. Interestingly, we found that butyrate inhibits the growth of MG-63 osteoblastic cells at concentrations higher than 4 mM, suggesting their roles in bony destruction and impairment of periodontal/periapical bone healing response.

The toxicity of butyric acid is associated with its influence on cell cycle progression. Intriguingly, we discovered that butyrate induced G2/M cell cycle arrest at higher concentration. In addition, butyrate induced apoptosis of MG-63 cells at higher concentrations. This may partly justify the decrease of viable cell number of MG-63 cells after exposure to butyrate. The G0/G1 and G2/M cell cycle progression is tightly controlled by a number of proteins such as cdc2, cyclin B1, and p21 [14,23,24]. During cell mitosis, cyclin B1 may form complex with cdc2 that can be dephosphorylated at T14 and Y15 by cdc25C phosphatase leading to full activation of its cdc2 Ser/Thr kinase activity and cell cycle progression [25–27]. However, limited information is known regarding the effect of butyrate on cell-cycle related genes of osteoblasts. Interestingly, we found that butyrate down-regulates cdc2, and cyclinB1 gene expression at transcriptional level. Moreover, the protein levels of cdc2 and cyclinB1 in MG-63 cells were also suppressed, whereas p21, p27 and p57 were stimulated after exposure to butyrate. Accordingly, butyrate, as an inhibitor of histone deacetylation, inhibits the growth of liver cancer cells via down-regulation of cdc2 and cyclin E and cyclin B1 in oral cancer cells [28,29]. In addition, three cyclin-dependent kinase inhibitors–p21, p27 and p57 may affect G0/G1 and G2/M cell cycle progression by inhibition of cdc2/cylin B1 and cyclin D/Cdk4/6. They also play important roles in cellular apoptosis, senescence, differentiation, cell motility and migration [30,31]. In this study, the induction of p21, p27 and p57 by butyrate may partially explain its growth inhibitory and apoptotic effects to MG-63 cells. However, butyrate (0.1 mM) has been shown to stimulate osteopontin, bone sialoprotein expression and mineralized nodule formation in healthy human osteoblasts, suggesting a role in bone differentiation [32]. Further studies are necessary to clarify the role of butyrate on differentiation, cell motility and migration of MG-63 osteoblastic cells.

In this study, butyrate also suppresses the type I collagen expression of MG-63 cells. Extracellular matrix proteins such as fibronectin and type I collagen have been shown to activate integrin receptors and down-stream signaling molecules to regulate survival, cell cycle progression, gene expression and matrix mineralization of osteoblasts [33]. The secretion of butyrate from periodontal/periapical microorganisms may thus impair bone repair by inhibition of type I collagen production. However, butyrate is also found to stimulate prostaglandin E_2_ (PGE_2_) production, cyclooxygenase (COX) expression, to regulate PGE_2_ receptors—EP1 and EP2 receptor, expression and the stimulation of collagen and osteopontin expression in ROS17/2.8 osteoblasts [34]. The precise reasons for this contrast result are not known and await further investigation.

The above toxic effects of butyrate on MG-63 cells may be associated with ROS production. Inducing cytotoxicity, genotoxicity and inflammatory mediators’ release by a number of chemicals such as cadmium, areca nut, resin monomers and camphorquinone are associated with cellular ROS production [12,17,20,35]. The sources of ROS can be derived from host cells and microorganisms such as *Porphyromonas gingivalis* and *Enterococci faecalis* [36–38]. Over-production of ROS may reduce cellular glutathione, damage DNA, proteins and lipids, to activate check-point kinases and control the cell cycle- and apoptosis-related genes [39,40]. Lately redox injectable gel has been effectively used to scavenge ROS and suppresses alveolar bone resorption in rat periodontitis models [41], suggesting the role of ROS in periodontal/periapical bony destruction. Intriguingly, ROS levels in MG-63 cell are elevated after exposure to butyrate in this study. Stimulation of ROS production may be involved in the toxicity of butyrate and the progression of bone destruction. These results indicate that accumulating and generation of butyrate in the periodontal and root canal biofilms may stimulate inflammatory mediators or deregulate host’s defense, contributing to periodontal or periapical bony destruction.

In conclusion, the results of this study indicate that butyrate generated by periapical/periodontal microorganisms exhibits cytostatic effect of osteoblastic cells probably via inhibition the cell cycle related genes, such as cdc2, and cyclinB1, as well as the induction of p21, p27 and p57 leading to cell cycle arrest and apoptosis. Butyrate also suppresses type I collagen expression. These events may impair the bone tissue repair and regeneration. These toxic effects of butyrate may be related to ROS production and contributes to the pathogenesis of periodontal/periapical tissue destruction.

## Supporting Information

S1 Table Effect of butyrate on type I collagen expression (fold of control) of MG-63 cells. The protein expression of western blot results was analyzed by Image J analysis.
(DOCX)

## References

[1] Nishihara T, Koseki T (2004). Microbial etiology of periodontitis. Periodontol 2000 36: 14–26. 10.1111/j.1600-0757.2004.03671.x 15330940

[2] Uematsu H, Sato N, Hossain MZ, Ikeda T, Hoshino E (2003) Degradation of arginine and other amino acids by butyrate-producing asaccharolytic anaerobic Gram-positive rods in periodontal pockets. Arch Oral Biol 48: 423–429. 12749914 10.1016/s0003-9969(03)00031-1

[3] Niederman R, Zhang J, Kashket S (1997). Short-chain carboxylic-acid- stimulated, PMN-mediated gingival inflammation. Crit Rev Oral Biol Med 8: 269–290. 9260044 10.1177/10454411970080030301

[4] Niederman R, Buyle-Bodin Y, Lu BY, Robinson P, Naleway C (1997) Short-chain carboxylic acid concentration in human gingival crevicular fluid. J Dent Res 76: 575–579. 9042080 10.1177/00220345970760010801

[5] Thomas LV, Suzuki K, Zhao J (2007). Bacterial pathogenesis and mediators in apical periodontitis. Braz Dent J 18: 267–280 18278296 10.1590/s0103-64402007000400001

[6] Niederman R, Buyle-Bodin Y, Lu BY, Naleway C, Robinson P, Kent R (1996) The relationship of gingival crevicular fluid short chain carboxylic acid concentration to gingival inflammation. J Clin Periodontol 23: 743–749 8877660 10.1111/j.1600-051x.1996.tb00604.x

[7] Pollanen MT, Overman DO, Salonen JI (1997). Bacterial metabolites sodium butyrate inhibits epithelial cell growth in vitro. J Periodont Res 32: 326–334. 9138199 10.1111/j.1600-0765.1997.tb00541.x

[8] Jeng JH, Chan CP, Ho YS, Lan WH, Hsieh CC, Chang MC (1999). Effects of butyrate and propionate on the adhesion, growth, cell cycle kinetics and protein synthesis of cultured human gingival fibroblasts. J Periodontol 70: 1435–1442. 10.1902/jop.1999.70.12.1435 10632518

[9] Chang MC, Tsai YL, Chen YW, Chan CP, Huang CF, Lan WC, et al (2013) Butyrate induces reactive oxygen species production and affects cell cycle progression in human gingival fibroblasts. J Periodontal Res 48: 66–73. 10.1111/j.1600-0765.2012.01504.x 22834967

[10] Morozumi A (2011). High concentration of sodium butyrate suppresses osteoblastic differentiation and mineralized nodule formation in ROS17/2.8 cells. J Oral Sci 53: 509–516. 22167038 10.2334/josnusd.53.509

[11] Kurita-Ochiai T, Fukushima K, Ochiai K (1995). Volatile fatty acids, metabolic by-products of periodontopathic bacteria, inhibit lymphocyte proliferation and cytokine production. J Dent Res 74: 1367–1373. 7560387 10.1177/00220345950740070801

[12] Ziech D, Franco R, Pappa A, Panayiotidis MI (2011) Reactive oxygen species (ROS)–induced genetic and epigenetic alterations in human carcinogenesis. Mutat Res, Fundamental & Molecular Mechanism Mutagenesis 711: 167–173. 10.1016/j.mrfmmm.2011.02.015 21419141

[13] Bertin G, Averbeck D (2006). Cadmium: cellular effects, modifications of biomolecules, modulation of DNA repair and genotoxic consequences. Biochimie 88: 1549–1559 10.1016/j.biochi.2006.10.001 17070979

[14] Galli C, Passeri G, Macaluso GM (2011). FoxOs, Wnts and oxidative stress-induced bone loss: new players in the periodontitis arena? J Periodont Res 46: 397–406. 10.1111/j.1600-0765.2011.01354.x 21332475

[15] Bartek J, Lukas J (2001). Pathways governing G1/S transition and their response to DNA damage. FEBS Lett 490: 117–22. 11223026 10.1016/s0014-5793(01)02114-7

[16] O’Connor PM (1997). Mammalian G1 and G2 phase checkpoints. Cancer Surv 29: 151–182. 9338101

[17] Chang MC, Lin LD, Wu MT, Chan CP, Chang HH, Lee MS, et al (2015) Effects of camphorquinone on cytotoxicity, cell cycle regulation and prostaglandin E2 production of dental pulp cells: Role of ROS, ATM/Chk2, MEK/ERK and hemeoxygenase-1. PLoS ONE 10: e0143663 10.1371/journal.pone.0143663 26658076 PMC4682794

[18] Chang MC, Wu HL, Lee JJ, Lee PH, Chang HH, Hahn LJ, et al (2004) The induction of prostaglandin E2 production, IL-6 production, cell cycle arrest and cytotoxicity in primary oral keratinocytes and KB cancer cells by areca nut ingredients is differentially regulated by MEK/ERK activation. J Biol Chem 279: 50676–50683. 10.1074/jbc.M404465200 15375172

[19] Chang HH, Guo MK, Kastan FH, Chang MC, Huang GF, Wang YL, et al (2005) Stimulation of glutathione depletion, ROS production and cell cycle arrest of dental pulp cells and gingival epithelial cells by HEMA. Biomaterials 26: 745–753. 10.1016/j.biomaterials.2004.03.021 15350779

[20] Jeng JH, Ho YS, Chan CP, Wang YJ, Hahn LJ, Lei D, et al (2000) Areca nut extract up-regulates prostaglandin production, cyclooxygenase-2 mRNA and protein expression of human oral keratinocytes. Carcinogenesis 21: 1365–1370. 10874015

[21] Lee PH, Chang MC, Chang WH, Wang TM, Wang YJ, Hahn LJ, et al (2006) Prolonged exposure to arecoline arrested human KB epithelial cells growth, regulatory mechanisms of cell cycle and apoptosis. Toxicology 220: 81–90. 10.1016/j.tox.2005.07.026 16413651

[22] Chang MC, Chen LI, Chan CP, Lee JJ, Wang TM, Yang TT, et al (2010) The role of reactive oxygen species and hemeoxygenase-1 expression in the cytotoxicity, cell cycle alteration and apoptosis of dental pulp cells induced by BisGMA. Biomaterials 31: 8164–8171 10.1016/j.biomaterials.2010.07.049 20673999

[23] Kurita-Ochiai T, Seto S, Suzuki N, Yamamoto M, Otsuka K, Abe K, et al (2008) Butyric acid induces apoptosis in inflamed fibroblasts. J Dent Res 87: 51–55. 18096893 10.1177/154405910808700108

[24] Taylor WR, Stark GR (2001). Regulation of te G2/M transition by p53. Oncogene 20: 1803–1815. 10.1038/sj.onc.1204252 11313928

[25] Muller GA, Engeland K (2010). The central role of CDE.CHR promoter elements in the regulation of cell cycle-dependent gene transcription. FEBS J 277: 877–893. 10.1111/j.1742-4658.2009.07508.x 20015071

[26] Le Breton M, Cormier P, Belle R, Mulner-Lorillon O, Morales J (2005) Translational control during mitosis. Biochimie 87: 805–811. 10.1016/j.biochi.2005.04.014 15951098

[27] Aressy B. Ducommun B (2008). Cell cycle control by the CDC25 phosphatases. Anticancer Agents Med Chem 8: 818–824. 19075563 10.2174/187152008786847756

[28] Wakabayashi K, Saito H, Kaneko F, Nakamoto N, Tada S, Hibi T (2005) Gene expression associated with the decrease in malignant phenotype of human liver cancer cells following stimulation with a histone deacetylase inhibitor. Int J Oncol 26: 233–239. 15586245

[29] Jeng JH, Kuo MY, Lee PH, Wang YJ, Lee MY, Lee JJ, et al (2006) Toxic and metabolic effect of sodium butyrate on SAS tongue cancer cells: role of cell cycle deregulation and redox changes. Toxicology 223: 235–247. 10.1016/j.tox.2006.04.033 16737765

[30] Tury A, Mairet-Coello G, DiCicco-Bloom E (2012). The multiple roles of the cyclin-dependent kinase inhibitory protein p57(KIP2) in cerebral cortical neurogenesis. Dev Neuobiol 72: 821–842. 10.1002/dneu.20999 22076965

[31] Rossi MN, Antonangeli F (2015). Cellular response upon stress: p57 contribution to the final outcome. Mediators Inflam 2015:259325. 10.1155/2015/259325 PMC4600511 26491224

[32] Katono T, Kawato T, Tanabe N, Suzuki N, Iida T, Morozumi A, et al (2008) Sodium butyrate stimulates mineralized nodule formation and osteoprotegerin expression by human osteoblasts. Arch Oral Biol 53: 903–909. 10.1016/j.archoralbio.2008.02.016 18406397

[33] Garcia AJ, Reyes CD (2005). Bio-adhesive surfaces to promote osteoblast differentiation. J Dent Res 84: 407–413. 15840774 10.1177/154405910508400502

[34] Iida T, Kawato T, Tanaka H, Tanabe N, Nakai K, Zhao N et al (2011) Sodium butyrate induces the production of cyclooxygenases and prostaglandin E2 in ROS 17/2.8 osteoblastic cells. Arch Oral Biol 56: 678–686. 10.1016/j.archoralbio.2010.12.013 21281931

[35] Chang MC, Chan CP, Chen YJ, Hsien HC, Chang YC, Yeung SY, et al (2016) Areca nut components affect COX-2, cdc2, and keratin expression as well as ADAM17, IL-1, PGE2 and 8-isoprostane production in oral keratinocytes: Role of reactive oxygen species, EGF and JAK signaling. Oncotargets 7: 16879–16894. 10.18632/oncotarget.7621 PMC4941357 26919242

[36] Battino M, Bullon P, Wilson M, Newman H (1999) Oxidative injury and inflammatory periodontal diseases: the challenge of anti-oxidants to free radicals and reactive oxygen species. Crit Rev Oral Biol Med 10: 458–476. 10634583 10.1177/10454411990100040301

[37] Waddington RJ, Moseley R, Embery G (2000). Reactive oxygen species: a potential role in the pathogenesis of periodontal diseases. Oral Dis 6: 138–151. 10822357 10.1111/j.1601-0825.2000.tb00325.x

[38] Szemes T, Vlkova B, Minarik G, Tothova L, Drahovska H, Turna J, et al (2010) On the origin of reactive oxygen species and antioxidative mechanisms in *Enterococcus faecalis*. Redox Rep 15: 202–206. 10.1179/135100010X12826446921581 21062535 PMC7067330

[39] Sarsour EH, Kumar MG, Chaudhuri L, Kalen AL, Goswami PC (2009) Redox control of the cell cycle in health and disease. Antioxid Redox Signal 11: 2985–3011. 10.1089/ARS.2009.2513 19505186 PMC2783918

[40] Ansteinsson V, Solhaug A, Samuelsen JT, Kalen AL, Goswami PC (2011) DNA-damage, cell cycle arrest and apoptosis induced in BEAS-2B cells by 2-hydroxyethyl methacrylate (HEMA). Mutat Res 723: 158–164. 10.1016/j.mrgentox.2011.04.011 21640196

[41] Saita M, Kaneko J, Sato T, Takahashi SS, Wada-Takahashi S, Kawamata R, et al (2016) Novel antioxidative nanotherapeutics in a rat periodontitis model: Reactive oxygen species scavenging by redox injectable gel suppresses alveolar bone resorption. Biomaterials 76: 292–301. 10.1016/j.biomaterials.2015.10.077 26559357

