# Supplementary material for: Effect of Butyrate on Collagen Expression, Cell Viability, Cell Cycle Progression and Related Proteins Expression of MG-63 Osteoblastic Cells
Source: PLoS One. 2016 Nov 28;11(11):e0165438. doi: 10.1371/journal.pone.0165438 (PMC5125573; doi:10.1371/journal.pone.0165438)
Supplement: S1 Table — The protein expression of western blot results was analyzed by Image J analysis. (DOCX) [file pone.0165438.s001.docx]

**Supplementary Table for Figure 4B.** Effect of butyrate (But) on collagen protein expression of MG-63 cells as analyzed by western blotting and Image J analysis. The expression of collagen I (band intensity) was divided by GAPDH (band intensity) for the control and butyrate-treated cells in 5 independent tests. Collagen I expression of butyrate-treated groups was expressed as fold of control (collagen 1/GAPDH = as 1). Results were expressed as Mean ± SE (n=5).

|  | control | Butyrate 1 | Butyrate 2 | (mM)  Butyrate 4 | Butyrate 8 | Butyrate 16 |
| --- | --- | --- | --- | --- | --- | --- |
| Test 1 | 1 | 1.13 | 0.5 | 0.39 | 0.22 | 0.32 |
| Test 2 | 1 | 0.40 | 0.26 | 0.20 | 0.05 | 0.06 |
| Test 3 | 1 | 0.84 | 0.74 | 0.57 | 0.54 | 0.46 |
| Test 4 | 1 | 0.95 | 0.55 | 0.48 | 0.46 | 0.09 |
| Test 5 | 1 | 1.13 | 0.81 | 0.64 | 0.49 | 0.23 |
| Fold of control  (Mean ± SE) | 1 | 0.89 ± 0.13 | 0.57 ± 0.09 | 0.45 ± 0.07 | 0.35 ± 0.09 | 0.23 ± 0.07 |
